# Sustaining the gains achieved by national neglected tropical disease (NTD) programs: How can we build NTD program country ownership and sustainability?

**DOI:** 10.1371/journal.pntd.0012211

**Published:** 2024-06-13

**Authors:** Yao Sodahlon, David A. Ross, Patrick O’Carroll, Carol McPhillips-Tangum, Joni Lawrence, Andie Tucker, Fabien Mpitu, Elizabeth Nicklas, Maura Tangum, Allison Goldberg, Marilyn Mainardi, Teshome Gebre, John Chiphwanya, Emilienne Epee, Piham Gnossike, Irène Megeh, Square Mkwanda, Ibarin Morou, Georges Barthelemy Nko’Ayissi, Laston Douglas Sitima, Ibrahima Socé Fall

**Affiliations:** 1 Mectizan Donation Program, Task Force for Global Health, Decatur, Georgia, United States of America; 2 Formerly with the Task Force for Global Health, Decatur, Georgia, United States of America; 3 Task Force for Global Health, Decatur, Georgia, United States of America; 4 Consultant to the Task Force for Global Health, Decatur, Georgia, United States of America; 5 Office of Corporate Responsibility, Merck & Co., Inc., Kenilworth, New Jersey, United States of America; 6 Neglected Tropical Disease Program, Ministry of Health, Lilongwe, Malawi; 7 Neglected Tropical Disease Program, Ministry of Health, Yaoundé, Cameroon; 8 University of Yaoundé, Yaoundé, Cameroon; 9 Ministry of Health, Public Hygiene and Universal Access to Care, Lomé, Togo; 10 Formerly with the Ministry of Health, Lilongwe, Malawi; 11 Department of Control of Neglected Tropical Diseases, World Health Organization, Geneva, Switzerland; London School of Hygiene and Tropical Medicine, UNITED KINGDOM

Preventable and treatable neglected tropical diseases (NTDs) affect more than 1 billion people globally [1]. These blinding and debilitating diseases negatively impact the quality of life among those affected and place a heavy burden on healthcare systems, particularly within low- and middle-income countries. As a result of effective public health interventions, such as mass drug administration (MDA) with sustained high coverage in endemic countries facilitated by generous drug donations and strong public–private partnerships, 50 countries had achieved elimination (as a public health problem or of transmission) of at least 1 NTD as of January 2024 [2]. More are on track to meet the targets for NTD elimination established by the World Health Organization (WHO) in its NTD Road Map: 2021–2030 [3].

Reaching NTD elimination goals is an important achievement. However, it does not mean that the incidence of these diseases has been permanently reduced to zero, interventions are no longer needed, or such diseases are “gone forever.” Victory lies in ensuring that these diseases never reemerge. As countries progress towards elimination goals and enter the post-MDA phase, there is a critical need for continued surveillance to detect any disease recurrence. Through post-MDA surveillance, ministries of health can be promptly alerted, and targeted disease control measures can be applied [4]. These activities conducted for the purpose of “Sustaining the Gain” are a critical component of a country’s public health capacity, essential for protecting the legacy of massive investments in NTD elimination. With significant progress made toward eliminating NTDs, we must ensure these hard-won gains are sustained.

Although the majority of endemic countries currently rely heavily on external funding and other resources from donors to support NTD elimination activities, countries are being called upon to increase domestic investment and ownership of their NTD programs [5–7]. There is a growing recognition among national NTD programs that they play an important role in creating sustainable programs to maintain progress. During a Coalition for Operational Research on NTDs (COR-NTD) meeting in 2019, NTD program managers expressed the need for technical assistance with domestic fundraising [8]. More recently, WHO conducted a survey with onchocerciasis program managers that highlighted their desire for technical assistance and training to effectively mobilize domestic resources to support NTD program activities [9]. There is clear, growing interest among national NTD program leaders in building capacity to ensure resources are available to sustain disease elimination activities, including ongoing interventions, monitoring, and evaluation.

In 2019, the Mectizan Donation Program and the Task Force for Global Health, with funding from Merck & Co, Inc. (known as MSD outside the United States of America and Canada), conducted an online survey of NTD program managers working with the Mectizan Drug Donation program on onchocerciasis and/or lymphatic filariasis to better understand country-level practices and challenges related to increasing domestic spending to sustain NTD interventions. The results of the survey underscored the importance of developing and implementing strategies to build country capacity to sustain NTD programs and progress toward disease elimination [10]. In 2022, Merck & Co., Inc. funded the Task Force for Global Health to engage 3 partner countries (Cameroon, Malawi, and Togo) to help develop strategies and tools to mobilize domestic resources for NTD elimination. The countries were selected based on criteria that included, but was not limited to, geographic location, NTD elimination status, and ability to provide NTD program data. The project culminated in a two-day, in-person workshop held in Addis Ababa, Ethiopia in 2023 attended by NTD program managers or coordinators from the 3 partner countries and representatives of the Task Force for Global Health.

The workshop was convened to address 5 objectives: (a) obtain additional input from country partners on tools and resources developed as part of the project; (b) explore how and when to use tools and resources; (c) learn from one another about how to identify, create, and act on domestic resource mobilization opportunities; (d) prepare to develop and implement country-specific evidence-based resource mobilization action plans; and (e) identify opportunities to sustain and expand domestic resource mobilization activities.

The first day of the workshop focused on sharing and learning from one another to develop strategies and identifying and engaging partners and audiences. The second day provided opportunities for partner countries to work together to create targeted and evidence-based messages and identify the next steps in domestic resource mobilization strategies. The second day also included a demonstration and user-experience (UX) testing with country representatives of an online guide developed by the Task Force for Global Health to make resources and tools available, as well as a discussion on opportunities to continue working and learning together to increase domestic investments needed to sustain the gains made by NTD elimination programs. The guide is available on the Task Force for Global Health website at www.taskforce.org/pathways/.

**Fig 1:**
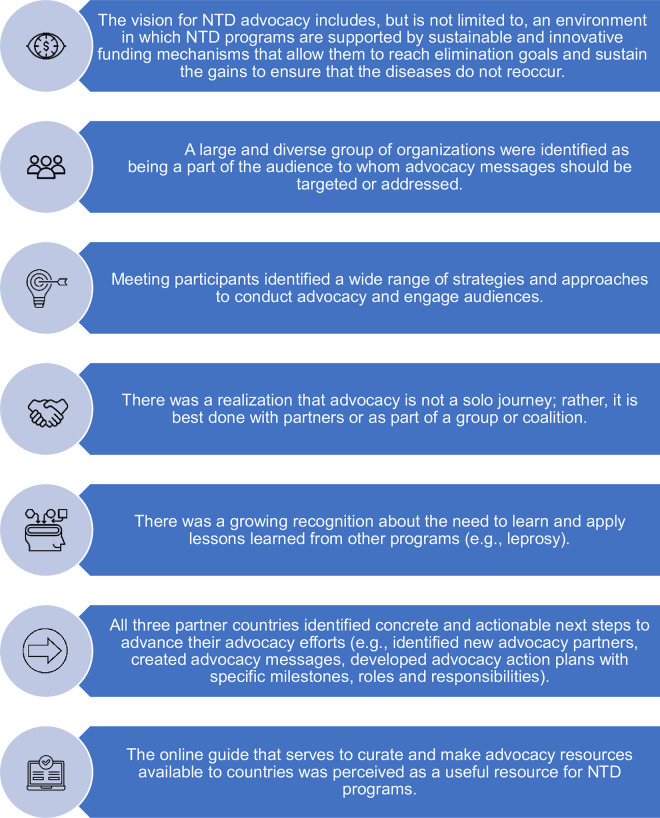
Themes and recommendations from the workshop.

Many insights emerged from the discussions. The workshop and information sharing helped support each country’s strategy towards influencing policy and NTD financing. Although the participants represented a variety of perspectives and positions, several common themes and agreed-upon recommendations emerged from the meeting (Fig 1). In addition to developing a collective vision for domestic resource mobilization, identifying new partners, and developing new strategies and approaches, workshop participants were very clear about the challenges and opportunities ahead. Despite the remarkable success that NTD programs have achieved, there is a history of these programs being siloed and far too modest to draw attention to their successes. Participants agreed that NTD programs might be one of the greatest public health success stories seldom told despite being hailed as cost-effective and a “best buy” in global health [11].

At the close of the workshop, participants unanimously agreed on the following key points: (a) NTD programs have been too passive in making community, business, and government leaders aware of the massive impact that NTD programs have had on their populations; (b) in this time of waning resources and competing priorities, it is absolutely critical that NTD programs champion their programs and progress to influence policy for NTD financing; (c) the global NTD community must consider the eventual transition from MDA treatment for NTDs toward a post-MDA phase to ensure resources for monitoring and surveillance are available; (d) this transition will require new partners, new roles for current partners, and more sophisticated strategies to sustain the gains made by NTD programs; and (e) the journey ahead should not be a solo one; rather, countries need ongoing opportunities to share and learn from one another to build capacity to effectively tell their story and secure commitment for the resources necessary to sustain the tremendous gains they have made over the past several decades.

In light of lessons learned from this project, we propose a concrete and actionable series of next steps and recommendations. First, the set of resource mobilization tools developed and vetted with country partners during the workshop will be made publicly available for use by other countries to enhance the knowledge, skills, and self-efficacy of NTD program managers and other health leaders to effectively increase domestic investment in NTD programs.

Second, a comprehensive and strategic approach is needed to build capacity within countries to enhance political will and engage business and civil society. Beyond having an awareness of the need to advocate for domestic resources and beyond having a strategy, program leaders and health officials need help in learning how to champion disease elimination efforts. Approaches, such as the communities of practice model and the model developed by the Global Partnership for Zero Leprosy to assist countries to review their current capacity and develop road map toward Zero Leprosy [12], can serve as examples to assist countries in developing strategies, building domestic resource mobilization skills and expertise, and putting strategies into action.

Third and perhaps most critical, a well-facilitated community of country partners supported by those with expertise in domestic resource mobilization is needed to catalyze effective results-driven country resource mobilization efforts. This underscores the need for global leadership—whether that be an agency, organization, or coalition—to serve as a convener and assume responsibility for assisting country-level capacity building to mobilize sustainable resources for NTD elimination. Experience tells us an entity (agency, organization, or coalition) is needed to build upon the momentum that has been created by this project and further support country learning and action.

We stand at a unique crossroad faced with an important opportunity. Organizations such as WHO are calling on countries to increase ownership and domestic spending on NTD programs [5], and countries are asking for guidance to help meet this goal. Now is the time for the global NTD community to unite and seize upon this opportunity by taking the necessary steps to build country-level capacity to ensure NTD services are included in health system priorities and budgets to sustain NTD programs’ march to true success and ensure that the remarkable progress made thus far is not lost.
